# Identification of p53-target genes in *Danio rerio*

**DOI:** 10.1038/srep32474

**Published:** 2016-09-01

**Authors:** Barbara Mandriani, Stefano Castellana, Carmela Rinaldi, Marta Manzoni, Santina Venuto, Eva Rodriguez-Aznar, Juan Galceran, M. Angela Nieto, Giuseppe Borsani, Eugenio Monti, Tommaso Mazza, Giuseppe Merla, Lucia Micale

**Affiliations:** 1 Medical Genetics Unit, I.R.C.C.S. “Casa Sollievo della Sofferenza” Hospital, San Giovanni Rotondo, 71013, Italy; 2 PhD Program, Molecular Genetics applied to Medical Sciences, University of Brescia, Brescia, 25123, Italy; 3 Bioinformatics Unit, I.R.C.C.S. “Casa Sollievo della Sofferenza”, San Giovanni Rotondo (FG), Italy; 4 Department of Molecular and Translational Medicine, University of Brescia, Viale Europa 11, 25123, Brescia, Italy; 5 Instituto de Neurociencias CSIC-UMH, Av. Ramon y Cajal s/n 03550 Sant Joan d’Alacant, Spain

## Abstract

To orchestrate the genomic response to cellular stress signals, p53 recognizes and binds to DNA containing specific and well-characterized p53-responsive elements (REs). Differences in RE sequences can strongly affect the p53 transactivation capacity and occur even between closely related species. Therefore, the identification and characterization of a species-specific p53 Binding sistes (BS) consensus sequence and of the associated target genes may help to provide new insights into the evolution of the p53 regulatory networks across different species. Although p53 functions were studied in a wide range of species, little is known about the p53-mediated transcriptional signature in *Danio rerio*. Here, we designed and biochemically validated a computational approach to identify novel p53 target genes in *Danio rerio* genome. Screening all the *Danio rerio* genome by pattern-matching-based analysis, we found p53 RE-like patterns proximal to 979 annotated *Danio rerio* genes. Prioritization analysis identified a subset of 134 candidate pattern-related genes, 31 of which have been investigated in further biochemical assays. Our study identified *runx1, axin1, traf4a, hspa8, col4a5, necab2,* and *dnajc9* genes as novel direct p53 targets and 12 additional p53-controlled genes in *Danio rerio* genome. The proposed combinatorial approach resulted to be highly sensitive and robust for identifying new p53 target genes also in additional animal species.

The intricate pattern of gene regulation involves the concerted action of both *cis*- and *trans*-acting transcription factors which bind to short nucleotide regulatory sequences in DNA modulating the expression of physically adjacent or very distant, even located on different chromosomes, coding sequences. Transcriptional networks evolution depends also on a number of modifications of master regulators (*trans*-evolution) or changes in *cis*-regulatory sequences (*cis*-evolution). Among sequence-specific transcription factors, p53 is a master regulator that, in response to stress conditions, coordinates the expression of a large set of target genes involved in a number of complex regulatory networks, which fulfil several important biological functions, such as apoptosis^1^, cell cycle regulation^2^, senescence^3^, cell differentiation, motility, and migration^4^,^5^,^6^,^7^,^8^. As a consequence of the cellular homeostasis and microenvironment variations, the level of p53 protein accumulation, the ability to interact with other proteins, and DNA binding activity and specificity were all finely tuned.

As a transcription factor, p53 recognizes its target genes by binding to a specific double-strand DNA sequence or response element (RE) composed of two palindromic, decameric half-sites of the general form: RRRCWWGYYY (where R stands for purine, Y for pyrimidine, W for adenine or thymine) separated by a 0–15 bp long spacer^9^. On the basis of X-ray crystallography of the p53–DNA complex, the 6 nucleotides 5′-RCWWGY-3′ are the most critical ones, which come into close contact with the core DNA-binding domain of the p53 protein^10^. p53 REs can be located anywhere within its target gene locus^9^, even if most of them are commonly found in the promoter region at varying distances upstream the transcription starting site (TSS), as it is the case of *p21* and *noxa* genes^11^.

Differences in RE sequences can strongly impact on the p53 transactivation capacity and occur even between closely related species^12^,^13^,^14^,^15^,^16^. For instance, human and mouse p53 proteins share a high sequence similarity and exhibit analogous biochemical functions and transactivation activity, while they diverge evolutionarily, both in terms of the p53 binding site (BS) sequences and, as a consequence, in terms of the p53-transcriptional network^15^,^17^,^18^. Therefore, the identification and characterization of a species-specific p53 BS consensus sequence and of the associated target genes may help to provide new insights into the evolution of the p53 regulatory networks across different species. Here, we designed and biochemically validated a computational tool to identify p53-target genes in *Danio rerio* (zebrafish). Our approach expanded the number of direct p53 target genes in this organism.

## Results

### *In silico* similarity search of putative p53-binding sites

A total of 3,033 hits for the RRRCWWGYYYN{0, 15}RRRCWWGYYY motif were found in the *Danio rerio* genome (Table S1), according to the PatMatch scanning analysis, with a substantially uniform pattern distribution among the 25 chromosomes (2.25 patterns per Mb, on the average). This analysis detected the 47% (1,420 out of 3,033) of the retrieved p53 DNA binding sites within repeat or transposable elements of *Danio rerio* genome. We ranged the spacer size from 0 to 15 bp and found a bi-modal frequency distribution, with peaks at 0 and 11 bp. A total of 1,277 gene-related patterns were found located in the ±5 Kb genomic neighbourhood of *Danio rerio* genes. In some cases, multiple genes resulted to be close to the same pattern or multiple patterns were found in the proximity of the same gene (Table 1 and S2). Particularly, 1,099 patterns were uniquely mapped to distinct genes, while 178 were proximal to more than one gene. Totally, this analysis identified 984 pattern-related genes including 979 protein-coding and 5 miRNAs. 140 out of 984 *Danio rerio* genes were associated to more than one pattern. Such genes as *arl13a*, *cetn3*, *rnf150a*, *runx1*, *zgc:136572*, *zgc:158766* have 10 or more motifs within their neighbourhoods. 472 out of 984 genes were positioned on the same genomic strand of their corresponding patterns (Table 1). All the genomic features of the found patterns are described in Tables S1 and S2.

Thus using the prioritization criteria (see Material and Methods section) (Table S3), along with the information about tissue specificity and the position of the patterns relative to the TSS, we selected a subset of 134 candidate motifs (Table S4).

As a preliminary investigation, we searched human orthologs for 134 prioritized zebrafish genes (Table S4) and checked their expression level into p53-related public datasets (Gene Expression Atlas 3.0, access: June 2016, https://www.ebi.ac.uk/gxa/home) (Table S5). A considerable proportion of Dr-retrieved gene/human-gene sets (53%, 73%, and 15,7% for p53, p53 and cancer, doxorubicin condition queries, respectively) was reported to be under- or over-expressed according to different experimental conditions suggesting their possible role as putative p53-controlled genes.

### p53-binding sites *in vitro* validation

We randomly selected 10 out of 134 predicted *Danio rerio* BSs, located at varying distances from the TSS of the related gene, for further *in vitro* validation (Table 2 and Table S6).

**Figure 1:**
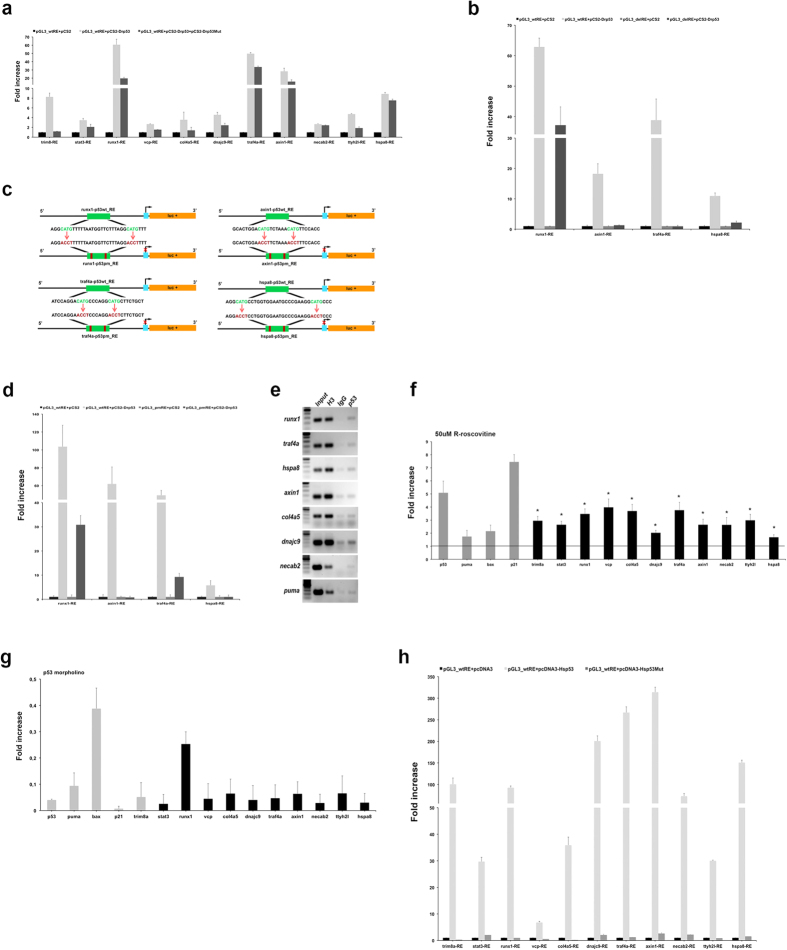
*Danio rerio* genes-embedded p53 BS show p53–dependent transactivation. **(a)** p53-null H1299 cells were cotransfected with reporter constructs carrying the predicted p53 RE of selected genes (pGL3_wtRE), *Dr*p53 expression vector (pCS2-*Dr*p53), an N-terminal truncated *Dr*p53 isoform, Δ1–113 *Dr*p53 (pCS2-*Dr*p53Mut), or pCS2 as a negative control. A vector containing *trim8a*-RE was used as a positive control. **(b)** p53-null H1299 cells were cotransfected with reporter constructs carrying or lacking the p53 REs (pGL3_wtRE or delRE of *runx1, axin1, traf4a,* and *hspa8,* respectively) and pCS2-*Dr*p53 or pCS2 as a negative control. **(c)** Schematic representation of pGL3 vectors carrying wild type (pGL3_wtRE) or mutated CWWG p53 RE (pGL3_pmRE, pm: point mutation) of *runx1, axin1, traf4a*, and *hspa8* genes. **(d)** p53-null H1299 cells were cotransfected with reporter carrying the wild type or mutated CWWG p53 REs of analyzed genes (pGL3_wtRE or pGL3_pmRE) and pCS2-*Dr*p53 or pCS2 as a negative control. **(e)** ChIP analyses were carried out with anti-p53 antibody on 24 hpf embryos. The p53 RE in *puma* is the positive control, H3 (line 3) and IgG (line 4) are the positive and negative controls of the immunoprecipitation, respectively. Loading marker (line 1). The data presented are representative of three independent experiments. **(f,g)** qPCR analyses were carried out on ten p53 pattern-related genes in 54 hpf embryos incubated with 50 *μ*M R-roscovitine **(f)** and in 30 hpf embryos injected with p53 MO **(g)**, compared to untreated embryos (horizontal black line). The data were normalized to the expression of *topI.* The expression of *bax*, *p21*^22^,^61^, *puma*^62^ and *trim8a*^19^ was measured as positive controls (grey bar). Bar represents the average of three independent experiments ± standard error. *P < 0.05. **(h)** p53-null H1299 were cotransfected with reporter constructs carrying the predicted genes p53 REs (pGL3_wtRE) and human p53 expression vector (pcDNA3-Hsp53) or transactivation defective mutant p53R175H (pcDNA3-Hsp53Mut) or empty control vector (pcDNA3) as a negative control. A vector containing *trim8a* p53 RE was used as a positive control. **(a,b,d,h)** Luciferase activities were normalized to the level of control *Renilla* luciferase. Each bar represents the average of three independent experiments ± standard error.

First, to assess whether *in silico* candidate patterns may drive the *Dr*p53-mediated transactivation or repression, we performed a luciferase-based assay. Short genomic regions harbouring selected *Dr*p53 BSs associated to 10 genes (Table 2 and Table S6) were cloned in luciferase reporter plasmids, and transfected into the human p53-deficient cell line H1299^19^ along with vectors expressing wild-type *Dr*p53, or an empty vector as a negative control. All amplified genomic fragments contain only one predicted p53 BS, but the region associated to *runx1* gene that includes four p53 BSs. A vector containing *trim8a* p53 REs was used as a positive control^19^. We found that all putative BSs mediate p53-responsiveness (Fig. 1a), although with different strength. Particularly, reporter gene activation mediated by *Dr*p53 was stronger for the p53 BSs potentially associated to *runx1* (60 folds), *axin1* (25 folds), *traf4a* (50 folds), and *hspa8* (9 folds) genes, when compared to the control. To test whether the activation was dependent on functional p53, we used an N-terminal truncated p53 isoform, Δ1–113 *Dr*p53 (pCS2-*Dr*p53Mut), which interferes with the p53 functions^20^. Consistent with the effect of this truncated form of p53, co-transfection of both full-length *Dr*p53 and Δ1–113 *Dr*p53 constructs in H1299 cells result in a luciferase activity decrease for all analysed BSs, when compared to transfection with full-length *Dr*p53 protein only (Fig. 1a).

Next, we generated mutant vectors carrying the deletion of BSs for those genomic fragments that exhibited strong reporter gene activation. As showed in Fig. 1b, *Dr*p53 was unable to activate the luciferase reporter gene for all constructs, except for p53-delRE-*runx1,* which was still responsive to the action of p53, likely because this construct still maintains three out of four predicted BSs. To define whether the conserved p53 RE CWWG core motif is necessary for reporter activation in *Danio rerio* as well as in human, we performed luciferase assays by using reporter vectors carrying nucleotide changes in the CWWG consensus (Fig. 1c). The results showed that replacing the core CWWG of *runx1*, *axin1*, *traf4a*, and *hspa8* p53 REs with ACCT sequence the responsiveness to p53 was dramatically reduced (Fig. 1d). Taken together, these results revealed that the analysed *Danio rerio* p53 BSs are functionally active, being each of them capable of conferring p53-dependent transcriptional activation on a luciferase reporter gene. Accordingly to the human p53 RE consensus, the core CWWG motif has a fundamental role in determining p53 activation function.

In order to confirm the functionality of these p53 BSs we investigated the native binding of endogenous p53 to the genomic locations where we have identified putative p53 BS. Chromatin immunoprecipitation in wild type 24 hour *Danio rerio* embryos (Fig. 1e) confirms that p53 binds to the *runx1*, *traf4a*, and *hspa8* p53 REs (ChIP). We also observed a slight enrichment in the binding to *axin1* p53 RE with respect to the control, probably reflecting the low level of *axin1* expression at this developmental stage. Additionally, we detect clear enrichment of p53 in the *col4a5*, *dnajc9,* and *necab2* p53 REs. This result indicates that the described p53 REs can act as enhancers through direct binding of p53 in the predicted binding sites, confirming that these seven genes are direct targets of p53. As a positive control for ChIP we analysed the binding of p5*3* to the p53-responsive element of a previously reported *Dr*p53 target gene, *puma*^21^. Conversely, we did not find any direct p53 binding in the *stat3*, *vcp*, and *ttyh2l* p53 RE.

To verify if the genes embedding the p53 BSs are *per se* responsive to the action of p53, we profiled *in vivo* the expression of ten p53 RE associated genes on 54 hpf zebrafish embryos incubated for 16 hours in presence of 50 *μ*M R-roscovitine, a cyclin-dependent kinase inhibitor that can efficiently stabilize and activate the nuclear p53 in human and zebrafish cells^22^,^23^. We found that the transcript levels of the genes surrounding the ten p53 BSs were increased in embryos upon exposure to R-roscovitine, compared to untreated embryos (Fig. 1f). Consistently, we observed a reduced mRNA level for all 10 genes in *Danio rerio* embryos injected with p53 antisense morpholino^24^, when compared to uninjected embryos (Fig. 1g).

Moreover we evaluated the responsiveness to human p53 of tested *Dr* BSs. We observed that the human p53 protein is able to efficiently activate the reporter gene expression (Fig. 1h), while the transactivation defective mutant p53-R175H (pcDNA3-MutHsp53) was unable to bind and activate the reporter constructs. Overall, these results confirm that p53 REs were responsive to both human and *Danio rerio* p53 proteins.

### PWM-driven similarity search of Drp53-target genes and *in vitro* validation

Subsequently, by using an additional similarity-based motif-search computational strategy we identified a second list of candidate p53 pattern-related genes for *in vitro* validation.

**Figure 2:**
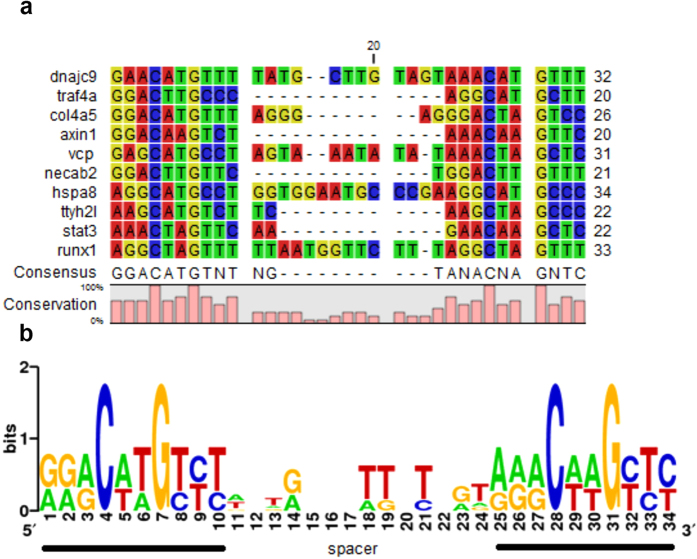
Nucleotide sequence alignment of the tested *Danio rerio* p53 binding sites. **(a)** Consensus sequence and the degree of conservation of each alignment site are shown on the bottom part of the alignment. **(b)** Consensus sequence logo has been created by using the WebLogo tool: http://weblogo.berkeley.edu/logo.cgi^63^. A total of 52 patterns were retrieved by MEME and RSAT motif similarity search (details shown in Supplementary Table S4).

We aligned the nucleotide sequences of the ten responsive elements initially validated by luciferase reporter assays (Fig. 2a,b, Table 2) calculating a new PWM (Table S7), which was finally inputted to two popular motif analysis resources, MEME and RSAT, and used as *seed* for an additional pattern similarity search. This strategy identified a group of 52 putative p53 pattern-related genes (Table S8), 21 of which were randomly selected for further *in vitro* validation (Table 3).

**Figure 3:**
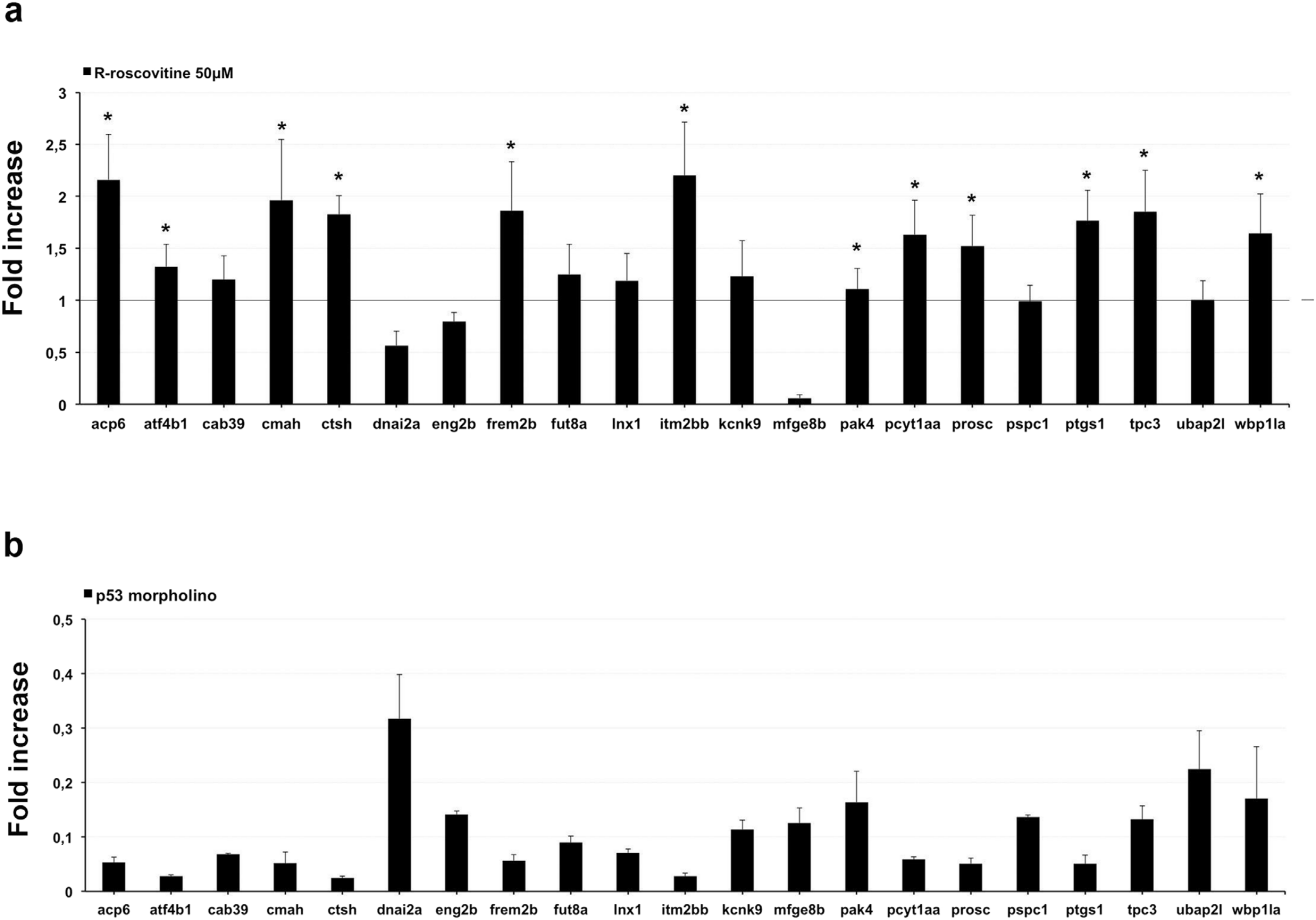
*In-vivo Dr*p53 exhibited transactivation potential on p53 REs-embedded genes. (**a)** qPCR was performed to measure the expression of twenty-one additional potential *Dr*p53 target genes on *Danio rerio* 54 hpf embryos incubated with 50 *μ*M R-roscovitine, compared to untreated embryos (horizontal black line). An increase of transcript levels in 12/21 analyzed genes was observed, compared to control embryos. **(b)** twenty-one candidate genes associated to *Danio rerio* p53 RE on 30 hpf embryos injected with p53 antisense morpholino compared to embryos uninjected, setting the threshold to one. A mRNA decrease of all twenty-one analysed genes was found. **(a,b)** The result obtained for all candidate genes was normalized to the expression of *top I*. Bar represents the average of three independent experiments and scale bars represent standard errors. *P < 0.05.

To verify whether such 21 genes are *per se* responsive to p53, we performed a gene expression profiling by qPCR in 54 hpf *Danio rerio* embryos incubated for 16 hours in presence of 50 *μ*M R-roscovitine (Fig. 3a) or in embryos injected with p53 antisense morpholino (Fig. 3b), finding a significant increase of transcript levels in 12 out of 21 analysed genes or an mRNA decrease of all genes, respectively, compared to untreated embryos (Fig. 3a,b).

**Figure 4:**
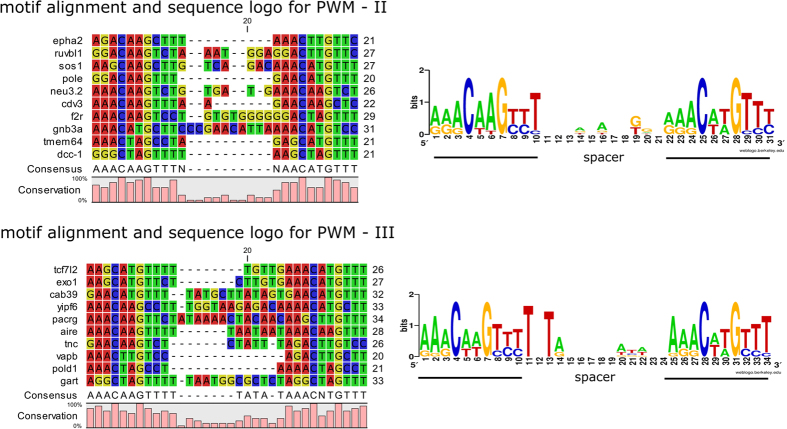
Generation of PWM-II and PWM-III matrices. Nucleotide sequence alignments and DNA logos deriving from the two additional motif sets (see Materials and Methods; details also in Tables S4 and S7).

**Figure 5:**
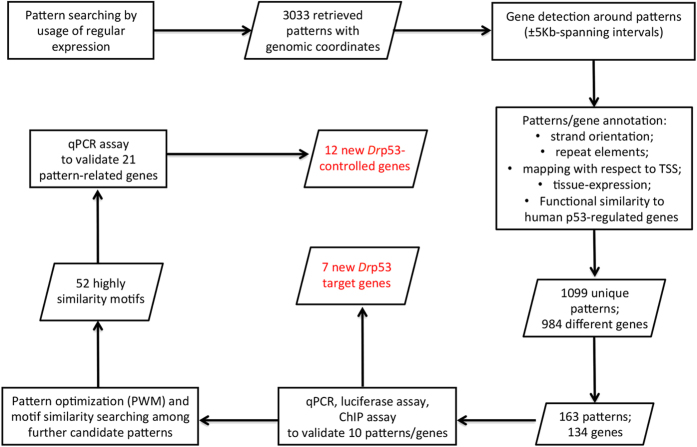
The experimental flowchart for the identification of the Drp53 pattern-related target genes. Rectangular boxes summarize the analysis steps described in the Material and Methods section. Parallelograms represent the data flow.

We also investigated the effects of our computational strategy for pattern prioritization by constructing two additional matrices and comparing results obtained. We randomly chose other two sets of sequences from the list of 134 candidate genes (Table S4) and created two further PWMs (Fig. 4 and Table S7). The selected patterns (Table S4, columns “PWM-II” and “PWM-III”) were analysed with the same combined strategy of motif similarity search, MEME and RSAT, applied to the first PWM, which was obtained from the first 10 validated motifs. Comparing the results obtained with the three PWMs, we found that 12 and 17 out of the 52 selected patterns (which are those with at least one significant similarity score calculated by the first PWM) were obtained when applying the two methodologies on the PWM-II, respectively. The analysis of the third PWM yielded, instead, 22 and 21 patterns from MEME and RSAT implementation, respectively (Table S8). The whole computational and biochemical analysis workflow is summarized in Fig. 5.

### Ingenuity Pathway Analysis

**Figure 6:**
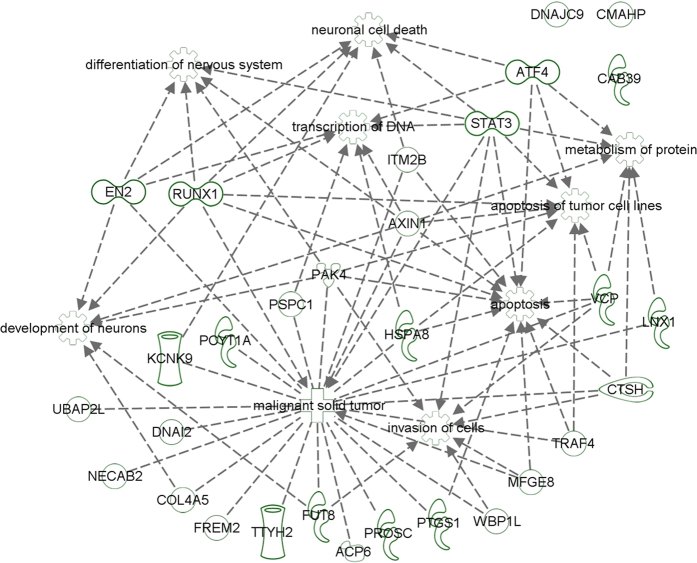
Functional network of 31 *Dr*p53 putative target genes. Arrows linking genes or connecting genes to biological processes indicate reported evidences of interaction or functional participation, respectively. An exhaustive legend of symbols is available at: http://ingenuity.force.com/ipa/articles/Feature_Description/Legend.

All 31 genes were systematically analyzed by Ingenuity Pathway Analysis in search of over-represented functional processes. As from Fig. 6, we verified a significant representation of neuronal (neuronal cell death, p-value = 2.86E-03; differentiation of nervous system, p-value = 5.81E-04; development of neurons, p-value = 1.05E-03), metabolic (metabolism of protein, p-value = 5.60E-03), gene expression-related (transcription of DNA, p-value = 4.51E-03) and cancer-related (apoptosis of tumour cell lines, p-value = 3.32E-03, apoptosis, p-value = 4.88E-03, malignant solid tumour, p-value = 1.01E-03, invasion of cells, p-value = 5.68E-04) processes.

The functional role of all the 31 investigated genes is summarized in Table S9, in which, for each gene, Gene Ontology Accession numbers and Terms have been reported.

## Discussion

The capacity of transcriptional regulatory machinery to quickly bring about changes in the gene expression pattern allows organisms to adapt to environmental and genetic changes. Transcription factor binding sites are generally known to evolve more rapidly than transcription factors themselves, playing a crucial part in the phenotypic divergence between and within closely related species^25^,^26^.

The binding of p53 with the DNA represents the crucial step by which the so-called “guardian of the genome” coordinates and regulates a wide set of downstream target genes. Because of the degenerate nature of the consensus p53 RE, changes in the level of p53 responsiveness have been observed and the rapid evolution of these regulatory elements determines variations in the p53 network between and within species^13^.

Therefore, the characterization of species-specific p53 BSs and target genes may help to provide insights into the evolution of p53 regulatory networks and to understand species-specific differences in several biological processes.

Here, we designed and biochemically validated a computational analysis that allowed us to identify novel p53 target genes in *Danio rerio* genome, an organism model widely used in human diseases and cancer, for whom little is known about the *Dr*p53-mediated transcriptional signature.

Screening all the *Danio rerio* genomic contexts by pattern-matching-based analysis, we found a total of 3,033 matching p53 RE-like patterns with 1,099 of them proximal to the annotated *Danio rerio* genes. In this analysis 47% (1,420 out of 3,033) of the retrieved patterns were detected within repeat or transposable elements in *Danio rerio* genome, as previously reported in ref. 19. In particular, the query pattern partially matched the elements belonging to the hAT-AC (30% of the total pool) and DNA (14%) repeat families or to the TE-X-4_DR transposable element (14%). These elements can promote the spread of p53 BSs, thereby contributing to the diversity of the p53 response between and within species, including p53-influenced processes, such as tumour formation, longevity, angiogenesis and fertility^27^,^28^,^29^.

Among the predicted gene-related patterns, we selected 10 binding sites and 10 matching pattern-related genes for *in vitro* validation using luciferase and qPCR assays, respectively, confirming their responsiveness to both *Dr*p53 and Hsp53. ChIP assays, performed in wild type 24 hours *Danio rerio* embryos, confirm that p53 directly binds to the *runx1*, *traf4a*, *axin1, hspa8, col4a5*, *dnajc9,* and *necab* p53 RE indicating that these genes can be considered as *bona fide* direct targets of p53. *Dr*p53 was not detected to *stat3, vcp*, and *tty2l* p53 RE that exhibited the lowest level of gene reporter activity for both *Dr*p53 and Hsp53.

The failure to detect direct binding of p53 to some of the p53 RE such as *stat3, vcp*, and *tty2l* might be due to technical issues such as low affinity sites, absence of cofactors or developmental stage as is shown by the low level of reporter activity for both Drp53 and Hsp53.

Notably, we observed a difference in reporter gene activation between human and zebrafish p53. This could be due to several reasons: i) *Dr*p53 REs were searched by using the human motif as starting query. It is thus conceivable that the selected *Dr*p53REs could be more specific for human p53 binding with respect to *Dr*p53; ii) the *Dr*p53 and Hsp53 are temperature sensitive proteins exhibiting their optimal activities at 28 °C and 37 °C, respectively, with *Dr*p53 displaying no transactivation or very weak activation at 37 °C^19^; iii) we carried out the luciferase assays in human cells cultured at 28 °C, which is not the standard condition for cell growth.

The validated pattern-gene pairs were subsequently used as *seed* for an additional similarity-based motif-search strategy that identified a second list of putative p53 targeted genes, among which we randomly chose 21 for further validation. We profiled their expression by qPCR both in *Danio rerio* embryos either silenced or enhanced for p53 expression, funding 12 additional potential p53 regulated genes.

Therefore, we cannot exclude the possibility that the other 9 genes, which are significantly deregulated in *Danio rerio* embryos that were injected with p53 antisense morpholino, but not in embryos treated with R-roscovitine, could be regulated by *Dr*p53.

Many factors could contribute to the differential transactivation to p53 observed in our assayed genes. Post-transcriptional and post-translational modifications of p53, recruitment of co-factors (co-repressors/chromatin-modifying factors), competition with transcription activator for DNA-binding site^30^, cellular and genomic context^31^, chromatin state, complexity of transcriptional regulation in living cells, features of the RE sequences, which take into account, i.e., of the variability of the spacer lengths, and the response variability upon each different p53 stimuli, as well as the R-roscovitine that interferes with the preferential activation of some p53-target genes, are possible causes. Regarding the latter point, we observed that the expression of *dnai2a* and *mfge8b* was down regulated in both R-roscovitine-treated and p53-antisense-injected embryos (Fig. 3a,b). Probably, a different p53-dependent transactivation of some genes could be observed in response to several DNA-damaging reagents activating specific p53 transcriptional programs.

Collectively, our analysis identified 7 novel p53 target genes: *runx1, axin1, traf4a, hspa8, col4a5, necab2,* and *dnajc9*. Interestingly, the p53 target genes identified in this study appear to involve broad functional classes and multiple gene families that are related, directly or indirectly, to the p53 pathway in human.

Human RUNX1 is a member of the RUNX transcription factor family that play essential roles in the vertebrate embryo by directly regulating transcription in a variety of developmental pathways including neurogenesis, bone development, and segmentation^32^. Runx1 has also an important role during hematopoiesis in fish as in mouse and human^33^. Interestingly, Masse *et al.* reported that a functional interplay between p63 and p53 regulates *RUNX1* expression in the control of the transition from proliferation to early differentiation in human keratinocytes^34^.

TRAF4 is a member of the TRAF family of adaptor proteins that mediate cellular signalling by binding to various members of the tumour necrosis family receptor superfamily and interleukin-1/Toll-like receptor super-family. The analysis of traf4-deficient mice revealed embryonic lethality underlying its potential importance during embryogenesis^35^. *TRAF4* is specifically regulated by p53 in response to overexpression of p53 by use of an adenovirus and stabilization of p53 in response to exposure to DNA-damaging agents. The murine *Traf4* genomic locus contains a functional p53 DNA-binding element located approximately 1 kilobase upstream of the translation start site. Overexpression of TRAF4 induces apoptosis and suppresses colony formation in multiple tumour cell lines suggesting a role for TRAF4 in determining cell fate in response to stabilization of p53^36^. *Danio rerio* ortholog traf4a shows a highly restricted expression pattern mainly in sensorial and neural cells, and in somites of *Danio rerio* embryos^37^.

Axin1 is a negative regulator of axis formation in the development of mouse embryos. Its deficiency leads to axis duplication^38^ and its overexpression blocked embryo axis formation in *Xenopus* and caused apoptosis in transgenic mice^39^,^40^. Accumulating data showed that Axin1 controls multiple important pathways, including the canonical Wnt pathway, JNK signaling, TGF-β signalling, and p53 activation cascade^41^,^42^,^43^.

A large variety of cellular functions have been attributed to heat shock protein HSPA8, most of them through its cooperation with cochaperones like its role as a clathrin-uncoating ATPase during clathrin-mediated endocytosis. HSPA8 is also a major actor in chaperone-mediated autophagy and its involvement in protein import into organelles or cellular compartments has been the focus of numerous studies. *HSPA8* has been identified as a p53-repressed target^44^. p53 binds a direct specific sequence on the *HSPA8* promoter followed by p53-dependent recruitment of Sin3B/HDAC1 co-repressor complex as well as hyper-methylation. On the other hand, *HSPA8* repression is critical for the functional activation of p53 because Hspa8 protein is known to antagonize the p53 nuclear localization by masking the nuclear localization signal sequence of p53^45^.

In conclusion, the approaches described here illustrate the great advantage of combining global p53 DNA-binding site *in silico* analysis and molecular assays, providing an efficient strategy to identify new p53 *Danio rerio* REs and target genes.

The identification and characterization of a *Danio rerio*-specific p53 BS consensus sequence and of the associated target genes may help to provide new insights into the evolution of the p53 regulatory networks in determining phenotypic diversity among species.

## Materials and Methods

### Pattern searching in *Danio rerio* genome

The first step of our computational strategy, which we have set up to identify the potential motifs of the p53 responsive elements in *Danio rerio,* encompasses the search of the human motif RRRCWWGYYYN{0, 15}RRRCWWGYYY in the *Danio rerio* genome. The build Zv9/danRer 7 version, July 2010, was considered as the reference genome (available at ftp://ftp.ensembl.org/pub/release-79/fasta/danio_rerio/dna/)^46^, from which we retrieved the genomic coordinates of the genes and of the repeat/transposable elements.

The pattern-matching tool initially used was PatMatch 1.2 (available at ftp://ftp.arabidopsis.org/home/tair/Software/)^47^. It implements an exact matching algorithm. It was run with this command line:

*perl patmatch.pl -n “RRRCWWGYYYN{0, 15}RRRCWWGYYY” Zv9.fasta.prepared > patmatch_Zv9_patterns.out,* where the argument “-n” defines the pattern, while the “prepared” fasta file consists of the input genomic sequences (strand ‘ + ’), manipulated as required by the tool. The expression “{0, 15}” indicates the spacer size. PatMatch produced a tabular output, containing all the nucleotide sequences that matched the pattern, together with the associated chromosomal coordinates. Results obtained by PatMatch were confirmed with p53scan 1.05 (available for download at www.rimls.nl/bioinfo/p53scan/)^48^. This tool implements a position weight matrix-based pattern-finding algorithm and uses a probabilistic approach that associates every matching site to a multinomial distribution (site-specific relative nucleotide frequencies, as defined by the p53scan PWM), rather than a single character state (one of the four nucleotides). It was run on the human p53 binding-site position weight matrix (PWM) accompanying the software package, by setting a spacer size ranging from 0 to 15. The command line was:

*python p53scan.py -i Zv9.fasta -s 0-15 > p53scan_Zv9_patterns.out*, where “Zv9.fasta” specifies the *Danio rerio* reference genome, while the “-s” parameter sets the allowable sizes of the p53 motif spacer.

Then, we explored the genomic context of each found pattern by choosing 5 Kb up and downstream following^48^,^49^,^50^ and focused only on contexts that included at least one annotated gene, according to the Zv9 reference gene coordinates. For each gene, we collected NCBI and Ensembl accession numbers, gene official symbol, CDS and exons coordinates, associated Gene Ontology terms via the Biomart query tool^51^. We thus annotated them in order to improve the sensitivity of the overall analytical strategy. First, we checked if putative target genes were located on the same genomic strand of the retrieved patterns. Considering also the distance from the TSSs, we labelled the patterns with the gene regions where they fell in (i.e., promoter, UTR, exon and intron).

Hence, the *Homo sapiens* orthologs of all the genes falling within the ±5 Kb up/down-stream genomic context of each pattern were searched in Ensembl 78, through Biomart, taking care of specifying that these were *Danio rerio* Ensembl Gene IDs. Homology relationships were obtained by extracting the gene trees of interest from the Ensembl Compara pre-computed phylogenies. The output consisted in a list of pairs of *Ensembl Human Gene ID* and *Homology Type*, comprising of only homolog genes with a one-to-one relationship. Among the different options of orthology predictions (“one2one”, “one2many”, “many2many”), we opted for the former, hypothesizing that such relationship should preserve the functional similarity of orthologs between *Danio rerio* and *Homo sapiens*.

Then, the resulting human ortholog genes were prioritized according to their functional proximity to 127 renowned p53-regulated human genes (Table S3), obtained from Riley *et al.*^9^. This set of 127 genes was basically used as *training* dataset for two gene prioritization bioinformatics tools: Endeavour^52^ and ToppGene^53^, which were run with default settings. We then considered the first 50 genes, which exhibited the most significant overall scores by Endeavour or ToppGene (Table S3).

In addition, information on gene expression was retrieved from the Zfin web resource^54^ by searching for tissue expression data for wild type organisms, from zygote to adult samples, through the “Expression” menu of the main page of the tool.

In summary, we first collected genomic and functional data related to each discovered “pattern-gene” pairs and then, we manually curated the selection of a limited number of them for *in-vitro*/*in-vivo* validation.

### Cloning of *Danio rerio* p53 binding sites, site-directed mutagenesis and dual-luciferase assay

Fragments of approximately 300–500 bp containing the predicted p53 BS were amplified by polymerase chain reaction (PCR) using a *Danio rerio* genomic DNA as template and then cloned into pGL3-basic vector (Promega). All constructs were verified by sequencing.

The human lung adenocarcinoma p53-null cell line H1299 was grown in DMEM medium supplemented with 10% fetal bovine serum (FBS; Life Technologies, USA) and 1% antibiotics mixture in a 37 °C incubator with 5% CO_2_. The reporter construct, p53 expression vector (pCS2-*Dr*p53 or pcDNA3-Hsp53) or mutant p53 expressing vector (pCS2-*Dr*p53Mut or pcDNA3-Hsp53Mut, an N-terminal truncated p53 isoform, Δ1–113 *Dr*p53, that lacks the transcription activation domain) or empty control vector, and pSV-Renilla (Promega) were co-transfected into p53-null H1299 cells plated in 96-well plates using Lipofectamine^®^ LTX with Plus™ Reagent (Life Technologies) according to the manufacturer’s instructions. Since it was previously reported that *Dr*p53 is a temperature sensitive protein^18^, cells were grown at 28 °C and 37 °C when transfected with pCS2-*Dr*p53 or pcDNA3-Hsp53 respectively. 48 h after transfection, cells were washed with PBS, lysed and assayed for both *Firefly* and *Renilla* luciferase activity using the Dual-GLO^®^ Luciferase Assay System (Promega) by a Glomax 96 microplate luminometer. *Firefly* luciferase activity was normalized to the *Renilla* luciferase activity for each transfected well, as reported in ref. 19. Constructs changing the p53 BS were generated by QuickChange II site-directed mutagenesis kit (Stratagene). The primer pairs used are listed in the Table S10.

### Chromatin immunoprecipitation

ChIP experiments were carried out following the EZ-Magna ChiP A/G Chromatin Immunoprecipitation Kit (Merck-Millipore) protocol essentially as described in ref. 21. Briefly, 30 dechorionated embryos (24 to 34 hour after fertilisation, AB strain) were fixed for 10 minutes in 1% formaldehyde, washed and sonicated in 300 μl of lysis buffer. Chromatin was immunoprecipitated with the anti-p53 antibodies kindly provided by Prof. G. Del Sal (University of Trieste, Trieste, Italy). The positive and negative controls were Rabbit anti-H3 (Abcam) and Rabbit anti-IgG (Diagenode) respectively. PCRs were performed with the Kapa 2G Fast HS (Kapa Biosystems) with the oligonucleotides indicated in Table S10. The data presented are representative of three independent experiments.

### Zebrafish care

Wild-type zebrafish (*Danio rerio*, AB type) embryos were obtained by natural spawning and were raised under standard conditions at 28 °C, with a 14-hour light/10-hour dark cycle in fish water (0.1 g/L Instant Ocean Sea Salts, 0.1 g/L sodium bicarbonate, 0.19 g/L calcium sulphate, 0.2 mg/L methylen blue, H_2_O) until the desired developmental stage was reached.

Embryos were staged according to Kimmel *et al.*^55^ and developmental stages of zebrafish embryos were expressed as hpf or dpf (hours or days post- fertilization, respectively) at 28 °C. This study has been approved by OPBA (Organismo Preposto al Benessere degli Animali) of the Ethics Committee of the University of Brescia. The use of the teleost fish *Danio rerio* – zebrafish - for the study of human disease has been approved by Ministry of Health. Animal experiments were carried out according to the relevant guidelines and regulations of the EU Directive 2010/63/EU.

### Morpholino injection

p53 Morpholino Oligo (MO) (GeneTools LLC, Philomath, OR, USA) targeting p53 zebrafish translation start site was used (GCGCCATTGCTTTGCAAGAATTG). p53 MO was diluted in 1x Danieau buffer (pH 7.6) and 2.5% tetramethylrhodamine dextran (Molecular Probes) as a tracer. 0.5 pmol of p53 expression–inhibiting morpholino were injected into the yolk of 1–2 cell stage embryos as previously reported^24^,^56^. Embryos were then raised until 30 hpf and processed for RNA extraction as described below. Injections were repeated three times; 100 embryos were injected and processed in every experiment. Non-injected embryos were used as negative control.

### Roscovitine treatment, RNA isolation and quantitative PCR

54 hpf *Danio rerio* embryos were incubated in fish water containing 50μM R-roscovitine (Calbiochem) or 0.1% (v/v) DMSO for 16 hours at 28 °C. Embryos were then grown for 8 more hours in absence of roscovotine, harvested, washed in PBS-DEPC and cryoconserved at −80 °C before performing the RNA extraction. Total RNA was extracted by using RNeasy^®^ Mini kit (Qiagen) and reverse transcribed using QuantiTect^®^ Reverse Transcription Kit (Qiagen), according to the manufacturer’s instructions. The expression levels of putative genes associated to p53 BS were examined by quantitative PCR (qPCR). The primer pairs were designed using the primer express software package^57^ with default parameters (Table S10). *top1* was used as housekeeping gene (Table S10). The reactions were run in triplicate in 10 μl of final volume with 10 ng of sample cDNA, 0.3 mM of each primer, and 1x Power SYBR Green PCR Master Mix (Termo Fisher Scientific-Applied Biosystems). Reactions were set up in a 384-well plate format with a Biomec 2000 (Beckmann Coulter) and run in an ABI Prism 7900HT (Termo Fisher Scientific-Applied Biosystems) with default amplification conditions. Raw Ct values were obtained using SDS 2.4.1 (Termo Fisher Scientific-Applied Biosystems). Calculations were carried out according to the comparative Ct method as reported in ref. 19.

### Identification of additional potential targets by pattern similarity search

The validated pattern-gene pairs were subsequently used as *seeds* for an additional similarity-based motif-search computational strategy. In detail, by using the CLC Sequence Viewer 7.5 program (http://www.clcbio.com/products/clc_sequence_viewer/), we aligned the nucleotide sequences of the responsive elements initially validated by luciferase reporter analysis. Therefore, since the consensus sequence of the p53 RE is degenerate, we used the aligned sequences to construct two new positional weight matrices (PWMs), one for each decamer, which were finally inputted to two popular motif analysis resources: MEME^58^ and the Regulatory Sequence Analysis Tools (RSAT)^59^. We run MAST 4.9.1 of the MEME suite with default parameters on both decamers, separately. MAST returned a series of top scoring input sequences, ordered by increasing E-values (i.e., the expected number of sequences in a random database of the same size that would match the motifs as well as the sequence, with 10 as cut-off value). In addition, we used the “matrix-scan QUICK” method of the RSAT with default parameters. The *Danio rerio*-specific nucleotide frequencies were used as background. As a result, we selected the best significant (i.e., p-value < 0.05) “input matrix vs. input sequence” alignments. The sites involved in the pairwise alignments were the alignment positions “1–10” or “24–34”. Finally, we crossed the top-ranked binding sites obtained with the two tools and made a second list of candidate patterns for *in-vitro* validation. This procedure was applied to two further matrices, built on 20 randomly selected patterns picked from the list of 52 “prioritized” patterns/genes. With this approach, we evaluated the degree of consistency of the results obtained with MEME and RSAT, when applied on the three PWMs, as well as the influence of the matrix-specific compositional features in the calculation of pattern similarity scores.

Validated genes were subjected to *in-silico* functional enrichment analysis by Ingenuity Pathway Analysis (IPA; QIAGEN, Redwood City, CA; www.qiagen.com/ingenuity). The procedure was based on the prior calculation of the activation z-scores, which were used to infer the activation states of the predicted biological functions. An enrichment score (Fisher’s exact test, p-value) was calculated to measure the overlap between observed and predicted functions^60^. A function was considered to be significantly over-represented by a set of genes if the corresponding p-value < 0.05.

## Additional Information

**How to cite this article**: Mandriani, B. *et al.* Identification of p53-target genes in *Danio rerio. Sci. Rep.* **6**, 32474; doi: 10.1038/srep32474 (2016).

## Supplementary Material

Supplementary Information

## Figures and Tables

**Table 1:** General features of the PatMatch patterns, with a focus on those detected in proximity to the *Danio rerio* genes.

Number of PatMatch patterns	3033
Number of gene-related patterns	1277
Number of unique gene-related patterns	1099
Genes (grouped by tipology)	979 protein-coding, 5 miRNA
Genes with more than one associated pattern	140
Number of reference transcripts	984
Number of patterns in the same orientation of genes	553
Number of genes in the same orientation of patterns	472
Proportion of patterns in 5′UTR	53/553
Proportion of patterns in upstream regions	73/553
Proportion of patterns within introns	194/553
Proportion of patterns within other gene regions	233
Proportion of gene-related patterns within repeats	254/553

**Table 2:** General features of the first 10 *Dr*p53 BSs: gene symbol, motif location, position with respect to the gene TSS, presence within repeat element and gene functional details are reported.

#	Gene	Chr	Pattern start	Pattern end	Position vs TSS	Overlap within repeat	Note
1	*hspa8*	10	30761068	30761101	exon9	No	Belonging to human P53 network.
2	*stat3*	3	16723031	16723052	intron22–23	No	High rank by both prioritization tools, but pattern is not located in canonical regions (within the 4–5^th^ intron).
3	*axin1*	3	43009715	43009734	intron5–6	Yes hAT-N54_DR	High rank by both prioritization tools; pattern in repeat element
4	*vcp*	5	43134399	43134429	intron8–9	Yes hAT-N52_DR	High rank by both prioritization tools; Pattern partially in repeat element
5	*runx1*	1	1067563	1067595	intron2–3	No	High rank by both prioritization tools; Multiple patterns in the region
6	*dnajc9*	12	10255976	10256007	upstream	No	Plausible pattern position with respect to TSS; expressed in central nervous system.
7	*traf4a*	15	14555737	14555756	5′UTR	No	Plausible pattern position with respect to TSS; expressed in central nervous system.
8	*necab2*	18	21197526	21197546	upstream	No	Plausible pattern position with respect to TSS ; expressed in central nervous system.
9	*ttyh2l*	3	52217686	52217707	upstream	No	Plausible pattern position with respect to TSS; expressed in neural tube.
10	*col4a5*	7	52559622	52559647	upstream	No	Plausible pattern position with respect to TSS; expressed in diencephalon.

**Table 3:** General features of additional 21 candidate *Dr*p53 BSs.

#	Gene	Chr	Pattern start	Pattern end	Position vs TSS	Overlap with repeat	Note
1	acp6	9	31514159	31514188	intron3–4	−	Pattern significantly similar to PWM.
2	atf4b1	6	214982	215012	upstream	−	Pattern significantly similar to PWM. Multiple patterns within the region.
3	cab39	15	38611426	38611457	upstream	−	Pattern significantly similar to PWM
4	cmah	4	2087845	2087866	upstream	−	Pattern significantly similar to the PWM (only with MAST tool)
5	ctsh	18	26206107	26206126	upstream		Pattern significantly similar to PWM (only with MAST tool). Within the top50 ranking genes according ToppGene.
6	dnai2a	12	37713739	37713769	upstream	−	Pattern significantly similar to PWM
7	eng2b	2	29688318	29688343	intron3–4	−	Pattern significantly similar to PWM. Expressed in whole organism, and, in particular, nervous system.
8	frem2b	15	33324401	33324435	upstream	−	Pattern significantly similar to PWM (only with MAST tool)
9	fut8	17	34073168	34073189	intron1–2	−	Pattern significantly similar to PWM. Expressed in whole organism.
10	lnx1	20	22699415	22699434	intron2–3	−	Pattern significantly similar to PWM
11	itm2bb	9	26365976	26366010	intron3–4	−	Pattern significantly similar to PWM. Expressed in whole organism.
12	kcnk9	19	5317791	5317810	intron1–2	−	Pattern significantly similar to PWM (only with RSAT).
13	mfge8b	25	19639397	19682265	upstream	−	Pattern significantly similar to PWM (only with MAST tool)
14	pak4	15	24783195	24817101	intron2–3	−	Pattern significantly similar to PWM (only with MAST tool). Within the top50 ranking genes according Endeavour.
15	pcyt1aa	2	9548952	9548972	upstream	−	Pattern significantly similar to PWM (only with MAST tool).
16	prosc	23	34273590	34273621	upstream	−	Pattern significantly similar to PWM
17	pspc1	1	26770983	26771015	upstream	−	Pattern significantly similar to PWM
18	ptgs1	5	68489605	68489628	upstream	−	Pattern significantly similar to PWM. Within the top50 ranking genes according ToppGene.
19	tpc3	11	31521673	31521704	intron2–3	−	Pattern significantly similar to PWM
20	ubap2l	19	8381258	8381282	upstream	−	Pattern significantly similar to PWM
21	wbp1la	13	28803993	28804014	upstream	−	Pattern significantly similar to PWM (only with MAST tool).

Gene symbol, motif location, position with respect to the gene TSS, and presence within repeat element are described. Considerations on pattern similarity to the PWM, tissue expression, and functional annotation are also reported.
